# Structural Equation Modeling Analysis of Factors Influencing Family Doctor Contracted Services Based on Survey Data from Changning District, Shanghai

**DOI:** 10.1155/2022/2648833

**Published:** 2022-06-23

**Authors:** Shanshan Liu, Zhiyun Jiang, Luan Wang, Jiaoling Huang, Tao Zhang, Chengjun Liu, Yimin Zhang

**Affiliations:** ^1^Pudong Institute for Health Development, Shanghai, China; ^2^Shanghai Jiao Tong University Affiliated Sixth People's Hospital, Shanghai, China; ^3^School of Public Health, Shanghai Jiaotong University School of Medicine, Shanghai, China; ^4^Shanghai Pudong New Area Geriatric Hospital, Shanghai, China; ^5^School of Social Development and Public Policy, Fudan University, Shanghai, China; ^6^Eye and Dental Diseases Prevention and Treatment of Pudong New Area, Shanghai, China

## Abstract

**Objective:**

Family doctors fulfill the role of gatekeepers in protecting residents' health with contracted services. Providing these valuable services involves multiple causes, relationships, and indirectly observable variables. This study used structural equation modeling to construct a dynamic model of the work of family doctors to provide a basis for incentives.

**Methods:**

This study used 2-year follow-up data from a survey of 294 family doctors in Changning District, Shanghai. Data were analyzed using confirmatory factor analysis and structural equation modeling. The measurement model and structural model were defined, identified, verified, integrated, and revised to identify the factors motivating family doctors to provide contracted services. A dynamic path for the family doctor contracted services model was established and eventually modified with six endogenous latent variables: cognition, environmental satisfaction, income satisfaction, support satisfaction, stability, and contracting performance, underpinned by 27 measurement variables.

**Result:**

The standardized regression coefficient of the effect of cognition on environmental satisfaction was 0.37 (*P* < 0.05) and the degree of variation interpretation was 0.14. The effect of cognition on income satisfaction was 0.54 (*P* < 0.05) and the degree of variation interpretation was 0.29. The effect of cognition on stability was 0.40 (*P* < 0.01), the effect of environmental satisfaction on stability was 0.12 (*P* < 0.05), and the effect of income satisfaction on stability was 0.22 (*P* < 0.05), all with a degree of variation interpretation of 0.369. Finally, the effect of stability on contracting performance was 0.51 (*P* < 0.05) with a degree of variation interpretation of 0.343.

**Conclusions:**

The degree of family doctors' understanding (cognition) of their own work largely determines their behavioral orientation and service effectiveness. These results raise the possibility of enhancing family doctors' work stability and improving the performance of contracted services by increasing the income of family doctors.

## 1. Introduction

To meet the medical and health needs of its people in different periods, China has been continuously reforming its medical and health system. China's current medical reform policy sets out to “maintain the basics, strengthen the grassroot level, and build the mechanism” with the aim of building an orderly and effective medical and health service system [1]. In 2015, “Guidance on promoting the construction of graded diagnosis and treatment system” was implemented to guide the construction of a graded diagnosis and treatment system on an internationally accepted model. Although different countries have different political, economic, and social systems, they have generally established a graded diagnosis and treatment system based on the first diagnosis at the grassroot level and orderly referral [2–4]. As currently practiced in China, the contracted service of family doctors is an important starting point for the implementation of graded diagnosis and treatment. After signing the contract, the patients can enjoy convenient measures such as a fast channel for medical treatment, extended prescription durations, and long-term prescriptions for chronic diseases, in addition to the care provided by family doctors who gain patients' trust and improve doctor–patient relations. Shanghai is the first region in China to carry out the reform of the family doctor system and has achieved outstanding results, such as the “1 + 1 + 1” contracting method (involving a family doctor, a secondary hospital, and a tertiary hospital), the innovation of contracted service fees, and intelligent information-based electronic file management [5, 6]. However, in the family doctor system, family doctors are the most important and basic resource [7]. Improvements in organization and management mechanisms are therefore needed to maximize the value of family doctors as healthcare service gatekeepers, even among those with the ability and motivation to provide optimal support and services [8].

We established a service value output framework covering three mechanisms, which we called “one body with two wings;” the body is the organizational management mechanism, and the two wings are the coordinated service mechanism (ability) and the compensation incentive mechanism (motivation). The valuable service output was the provision of an orderly and effective medical service system, with the family doctor acting as a healthcare and cost gatekeeper. To explore the factors that encourage family doctors to provide services that meet patient needs, this study used structural equation modeling (SEM) to consider multiple causes, relationships between multiple results, and variables that are not directly observable (latent variables). SEM is used to explore the relationship between sets of observable variables and latent variables by obtaining the covariance between these variables from matrix Σ to characterize these relationships. Matrix Σ generates a covariance matrix Σ' that is as close as possible to sample covariance matrix S. If the theoretical model is correct, then Σ' = Σ; therefore, the variance and covariance of the observed variables are functions of the model parameters [9, 10].

This study was based on the need to establish an orderly and effective medical service system, in line with the survey data from family doctors in Changning District, Shanghai. We used theoretical analysis and reasoning to establish a hypothetical conceptual model, with valuable contracted services as the core, and SEM for model verification and amendments. This family doctor dynamic model was initially constructed to provide a basis for decision-making about healthcare, health insurance, and pharmaceutical reforms.

## 2. Materials and Methods

### 2.1. Participants and Procedure

We obtained full-survey coverage of the general practitioners who worked as family doctors in Changning District, the pilot area for reform of the family doctor system in Shanghai. The participants were all family doctors who provided contracted services in community health service centers in Changning District.

We developed the survey instrument “Questionnaire of Family Doctor Working Status in Community Health Centers” based on the published literature [11–13]. The survey used an anonymous self-filled questionnaire comprising of four parts with 50 items, covering basic personal information (seven items), work competency (15 items), family doctors' perceptions (17 items), and job satisfaction (11 items). The research group was responsible for distributing and collecting the questionnaire. The first survey, conducted in December 2013, included 154 respondents, with 152 completed the valid questionnaires. A 2-year follow-up questionnaire survey was conducted in August 2016 and involved 143 respondents, with 142 valid questionnaires. After eliminating invalid questionnaires, the effective questionnaire recovery rate was 98.99%. The data were entered using the EpiData software by the parallel double-entry method.

In total, 294 family doctors were included in the survey, of whom 66.32% (191/288) were women and 48.08% (138/287) were aged 30–39 years. The most common education level was a bachelor's degree (75.69%, 218/288). Most respondents (78.52%, 223/284) held intermediate titles, and 66.55% (187/281) of them had worked for more than 4 years.

### 2.2. Model Design and Assumptions

A hypothetical conceptual model was developed drawing on the literature and then verified and revised using SEM to construct the family doctor dynamic path model.

#### 2.2.1. Methodological Description of SEM

SEM, also known as covariance structure modeling, is used to process complex multivariate research data [14]. It integrates measurements and analyses with a factor model known as the measurement model that describes the relationship between observable and latent variables. The causal model, called the structural model, describes the relationship between latent variables. The parameters that make up these two models are called measurement model parameters and structural model parameters. The measured variables in SEM, also called observed variables or manifest variables, are directly measured through questionnaires or experiments. These are the most basic elements of data analysis. Latent variables are inferred from the measured variables. Endogenous variables are those that are affected by other variables in the model, and exogenous variables are those that are not affected by any other variable in the model but affect other variables.

Examining the relationship between the traditional dependent variable and the independent variable, external variables must be independent because they are not affected by other variables. Internal variables are mostly used as dependent variables but may also be used as independent variables that affect others. The dependent and independent variables become mediators. Typically, in SEM, the external and internal variables in the causal model are the latent variables. The model is as follows:

(1) $$\begin{array}{c} y=\Lambda y\eta +\varepsilon ,\\ x=\Lambda x\xi +\delta ,\\ \eta =B\eta +\Gamma \xi +\zeta . \end{array}$$

#### 2.2.2. Building the Family Doctors' Dynamic Path model

*(1) Confirmatory factor analysis (CFA) of measurement models*. The family doctor job satisfaction model (model 1) composed of original observation indicators proposed using the theoretical design, including 3 exogenous latent variables and 16 measurement variables. The first exogenous latent variable was environmental satisfaction, with 9 measurement variables: office conditions, institutional equipment, medical technology systems, administrative support and logistics, organizational management, promotion, leadership qualities, interpersonal relationships, and information technology. The second exogenous latent variable was income satisfaction, with 4 measurement variables: income level, pay matching, welfare, and fairness of income. The third exogenous latent variable was collaborative diagnosis and treatment, community cooperation, and team support. Each measurement variable had a measurement residual (*e*), with *e*1–*e*16 in total. Each measurement variable was only affected by a single latent variable, resulting in 16 factor-loading parameters (*λ*1–*λ*16). Factor changes were allowed free estimation, resulting in 3 correlation coefficient parameters. A pairwise correlation was established between the exogenous latent variables.

To provide an estimate of the relationship between latent variables and observed variables, regardless of the potential endogenous or exogenous variables, it was necessary to fix the first factor of the observed variables for each latent variable. The first load parameters (Λ*y*, Λ*x*) were 1, as a reference for other load parameters, and the variance of the 3 potential variables was freely estimated.

The family doctor competence, cognition, contracting performance, and stability model (model 2) composed of 4 exogenous latent variables and 20 measurement variables. The exogenous latent variable competence had 5 measurement variables: knowledge ability, contract content, basic medical knowledge, chronic disease management, and job training. The exogenous latent variable cognition had 7 measurement variables: clear content, clear responsibilities, work value, ability, professional reputation, social status, and satisfaction in achievements. The exogenous latent variable contracting performance had 5 measurement variables: community first consultation, identity participation, voluntary signing, meeting demand, and improving efficiency. The exogenous latent variable stability had 3 measurement variables: prospects, job expectations, and career opportunities. There were also 20 measurement residuals (*e*), *e*1–*e*20. Each measurement variable was only affected by a single latent variable, resulting in 16 factor-loading parameters (*λ*1–*λ*20). Factor changes were allowed free estimation, resulting in 6 correlation coefficient parameters, and a pairwise correlation was established between the exogenous latent variables.

To estimate the relationship between the latent variables and observed variables, regardless of the potential endogenous or potential exogenous variables, it was necessary to fix the first factor among the observed variables for each latent variable. The first load parameters (Λ_*y*_, Λ_*x*_) were 1, as a reference for other load parameters, and the variance of the 4 potential variables was freely estimated.

*(2) Construction and verification of the structural equation integration model*. SEM was used to analyze the structural relationships between potential concepts within the model framework. This examined the relationship between family doctors' satisfaction, competence, cognition, contracting performance, and stability with other variables, considering the impact of the index construction (accumulation) and potential measurement errors.

A conceptual path map of a unified model with both measurement and structural equations (model 3) was drawn. Synthetic variables in this model did not appear in the form of measured variables but were used to estimate potential concepts through the model to control errors. The main advantage of this approach is that it is possible to estimate all parameters in the model at the same time, test the overall theoretical model, and observe the appropriateness of the data.

Measurement model assumptions were as follows:

1. Exogenous latent variable (*ξ*): cognition (*ξ*1) included 5 exogenous explicit variables (*x*1–5), measurement residuals (*e*1–*e*5), and the corresponding measurement error terms (*δ*1–5);
2. Endogenous latent variable (*η*): competence (*η*1) included 3 endogenous dominant variables (*y*1–3), measurement residuals (*e*6–*e*8), and the corresponding measurement error terms (*ε*1–3); environmental satisfaction (*η*2) included 9 endogenous dominant variables (*y*4–12), measurement residuals (*e*9–*e*17), and the corresponding measurement error terms (*ε*4–12); income satisfaction (*η*3) included 4 exogenous explicit variables (*y*13–16), measurement residuals (*e*18–*e*21), and the corresponding measurement error terms (*ε*13–16); support satisfaction (*η*4) included 3 exogenous explicit variables (*y*17–19), measurement residuals (*e*22–e24), and the corresponding measurement error terms (*ε*17–19); stability (*η*5) included 2 exogenous explicit variables (*y*20–21), measurement residuals (*e*25–*e*26), and measurement error terms (*ε*20–21); and contracting performance (*η*6) included 3 exogenous explicit variables (*y*22–24), measurement residuals (e27–29), and measurement error terms (*ε*22–24).

**Figure 1:**
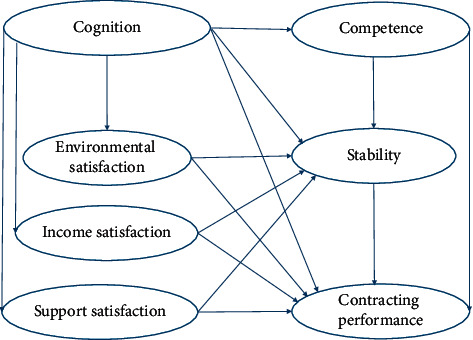
Hypothetical model path diagram of family doctor contracted services.

Structural model assumptions were as follows:

1. The endogenous latent variable competence (*η*1) was affected by the exogenous latent variable cognition (*ξ*1), and *e*30 was the interpreted residual *ζ*1;
2. The endogenous latent variable environmental satisfaction (*η*2) was affected by the exogenous latent variable cognition (*ξ*1), and *e*31 was the interpreted residual *ζ*2;
3. The endogenous latent variable income satisfaction (*η*3) was affected by the exogenous latent variable cognition (*ξ*1), and *e*32 was the interpreted residual *ζ*3;
4. The endogenous latent variable support satisfaction (*η*4) was affected by the exogenous latent variable cognition (*ξ*1), and *e*33 was the interpreted residual *ζ*4;
5. The endogenous latent variable stability (*η*5) was affected by the exogenous latent variable cognition (*ξ*1) and the endogenous latent variables competence (*η*1), environmental satisfaction (*η*2), income satisfaction (*η*3), and support satisfaction (*η*4), and *e*34 was the interpreted residual *ζ*5;
6. The endogenous latent variable contracting performance (*η*6) was affected by the exogenous latent variable cognition (*ξ*1) and the endogenous latent variables competence (*η*1), environmental satisfaction (*η*2), income satisfaction (*η*3), support satisfaction (*η*4), and stability (*η*5). *e*35 was the interpreted residual *ζ*6. The hypothetical model path of family doctor contracted services is shown in Figure 1.

### 2.3. Statistical Analysis

The data were input using the EpiData software (v.3.1; Redwood City, CA, USA). Statistical analyses used SPSS (v. 19.0; IBM SPSS, Armonk, NY, USA), and AMOS (v. 21.0; IBM SPSS, Armonk, NY, USA) was used for model testing. The analytical methods included ordinal regression methods and descriptive statistics (mean and standard deviation, or number and percentage), and the most approximate likelihood (ML) estimation was used in the SEM analyses.

## 3. Results

### 3.1. Analysis of the Family Doctor Job Satisfaction Model

**Figure 2:**
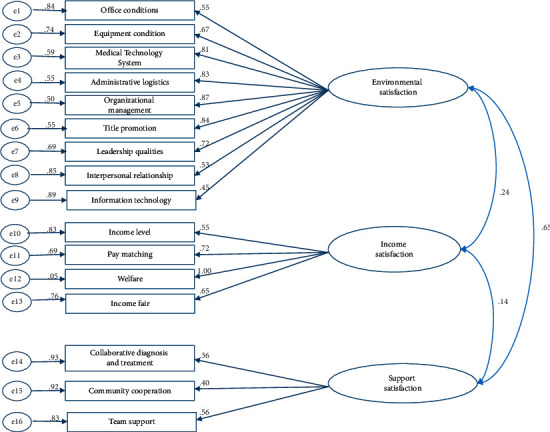
Family doctor job satisfaction measurement model (model 1).

Model 1 included 3 exogenous latent variables, environmental satisfaction, income satisfaction, and support satisfaction, with 9, 4, and 3 measurement variables, respectively (Figure 2).

After CFA, the standardized factor load values of interpersonal relationships and income level were close to 0.55 and those of community cooperation, collaborative diagnosis and treatment, and information technology methods were lower than 0.55; all the others were greater than 0.55. The multiple correlation squares (MCS) of measured variables were greater than 20% except for community cooperation and collaborative diagnosis and treatment (Figure 1). The overall goodness-of-fit index of model 1 was average, and the model was basically acceptable (Table 1).

### 3.2. Analysis of Family Doctor Competence, Cognition, Contracting Performance, and Stability Model

**Figure 3:**
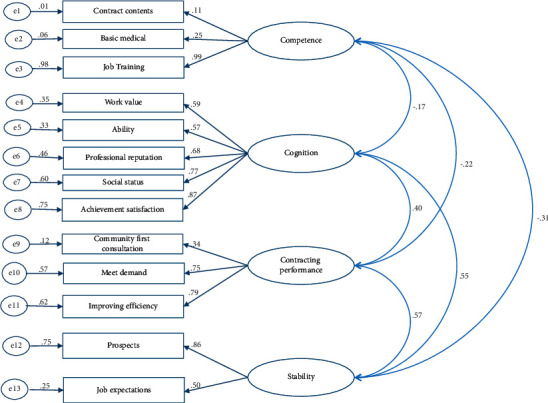
Family doctor competence, cognition, contracting performance, and stability revised model (model R2).

Model 2 included 4 exogenous latent variables, namely, competence, cognition, contracting performance, and stability, containing 5, 7, 5, and 3 measurement variables, respectively. After CFA of model 2, *λ* for all the exogenous latent variables for family doctor competence, except for job training, knowledge ability, and contract content, was higher than 0.55. The SMC of all measured variables was higher than 20%, except for job training, contract content, and knowledge ability. For most of the measurement variables of cognition, except for clear content and clear duty, *λ* was higher than 0.55. Similarly, the SMC score was higher than 30%, except for clear content and clear responsibility, which were both less than 20%. For all the elements of contracting performance, except for active signing, identification of participation, and community first consultation, *λ* was higher than 0.55. These 3 variables all had *λ* lower than 0.55, and SMC score was lower than 20%. For the exogenous latent variable stability, the SMC score of job expectations and development prospects was higher than 20%. Model 2 was therefore modified to model R2 to fit the results; latent variables with lower *λ* were deleted from the measurement variables. The fitting index of model R2 was significantly improved (Figure 3), and the estimated value of the standardized factor load (*λ*) was greater than 0.50, except for contract content, basic medical knowledge, and community first consultation. CFA was performed as given in Table 1. The fitting effect of model R2 was acceptable.

### 3.3. Analysis of the Dynamic Path of Family Doctor Contracting Services

**Figure 4:**
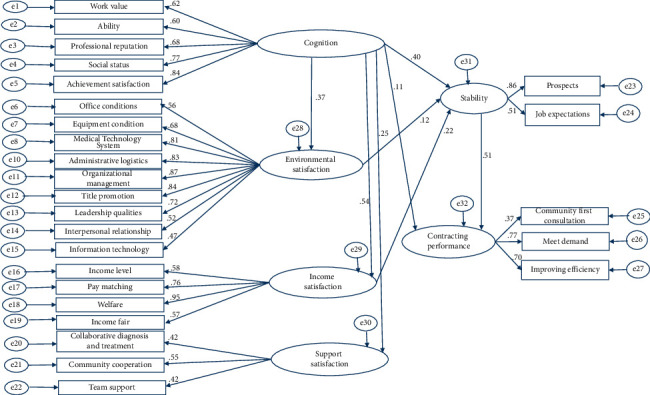
Dynamic path of the family doctor contracting service revised model (model R3).

The CFA fitting effect of model 3 showed that the *χ*^2^/d*f* index met the fitting requirements, and the root mean square error of approximation (RMSEA) of 0.092 was also within the acceptable range, but the overall fitting effect was average (Table 2). The parameter estimation results showed that the variances of the exogenous latent variables, the endogenous latent variables, the measurement error terms, and the interpreted residual terms were all positive. The sign of the measurement model coefficient was in line with theoretical expectations, the structural path coefficient (*γ* cognition on income satisfaction, *γ* cognition on environmental satisfaction, *γ* cognition on stability, *β* income satisfaction on stability, and *β* stability on contracting performance) was positive, and the differences were statistically significant (*P* < 0.05). However, we found that the structural path coefficient (*β* competency on contracting performance, *β* competency on stability, *β* support satisfaction on stability, *β* environmental satisfaction on contracting performance, and *β* income satisfaction on contracting performance) was negative, which was inconsistent with our theoretical assumptions. Model 3 was therefore modified to model R3 as the final model (Figure 4).

The standardized regression coefficient *γ* of the exogenous latent variable cognition (*ξ*1) on environmental satisfaction (*η*1) was 0.37 (*P* < 0.05) and the degree of variation interpretation (SMC) was 0.14. The standardized regression coefficient *γ* of cognition (*ξ*1) on income satisfaction (*η*2) was 0.54 (*P* < 0.05) and SMC was 0.29. The standardized regression coefficient *γ* for cognition (*ξ*1) on support satisfaction (*η*3) was 0.25 and SMC was 0.062. The standardized regression coefficient *γ* for cognition (*ξ*1) on stability (4) was 0.40 (*P* < 0.01) and SMC was 0.369. The standardized regression coefficient *γ* of cognition (*ξ*1) on contracting performance (*η*5) was 0.11, the standard regression coefficient *γ* of stability (*η*4) on contracting performance (*η*5) was 0.51 (*P* < 0.05), and the SMCs for both were 0.343. The standardized regression coefficient *γ* of environmental satisfaction (*η*1) on stability (*η*4) was 0.12. The standardized regression coefficient *γ* of income satisfaction (*η*1) on stability (*η*4) was 0.22 (*P* < 0.05). The standardization effect decomposition and significance of the structural model are given in Table 3.

## 4. Discussion

As an important starting point for resolving the contradiction between the people's growing needs for a better life and unbalanced and inadequate development, the Chinese medical reform aims to improve health and return the focus of medical care to the community. The construction of a graded diagnosis and treatment system in China involves a wide range of areas and is highly policy-oriented, long-term, and complex. The gradual improvement of the system also faces some problems. First, the professional and technical level of primary medical institutions is relatively low, making it difficult to win the trust of the people and affect implementation. Second, the two-way referral system is not perfect; the downward referral channel in particular is not smooth. Third, reform restricts the medical service projects provided by primary medical and health institutions, especially the contracted service projects of family doctors. Fourth, the unbalanced level of informatization affects the utilization and allocation of high-quality medical resources [15–17].

Family doctor contracted services are an important starting point for promoting the graded diagnosis and treatment system, as confirmed in other countries [18–21]. The National Health Service (NHS) in the United Kingdom established a primary healthcare network with general practitioners providing basic healthcare services and implemented a strict community first consultation and referral system. In the United States, the process of seeing a doctor is actually determined by the patients' form of insurance. But in fact, no matter what kind of medical insurance the patients choose, basically, every patient has a family doctor; moreover, family doctors are trained for 3 years of regular family medicine or internal medicine and are familiar with the treatment of common diseases and the mastery of guidelines. In Germany, training high-quality general practitioners is the most important way to implement hierarchical diagnosis and treatment. A strict general practitioner training system has been established, including 6-year training in school, 18-month trainee training,3-year general practice training, and lifelong medical continuing education. Canada implements a tiered medical system, including primary care, secondary care, and tertiary care, of which the primary care is provided by family doctors. Therefore, the ability and stability of family doctors directly affect the advancement of the hierarchical diagnosis and treatment system. By training general practitioners and promoting family doctors contracting to continuously improve doctors' diagnosis and treatment capabilities and increase service items, and augmenting medical equipment in general practice, implementing family doctor contracting services could gradually enhance grassroot service capabilities.

We conducted a preliminary study to understand the development process of the family doctor system in Changning District, Shanghai. Standardization of family doctors can optimize the service process, and the unification of family doctor service technical standards and specifications can effectively improve the service ability and quality of family doctors [22]. The establishment and innovation of service models is central to providing valuable general practice team services. Further promotion of the family doctor system requires building capabilities (collaboration platform) and motivation (medical insurance payment). Improvements in these two major mechanisms will be needed to sustain reform beyond the current development stage. The family doctor system in Changning District has undergone 5 stages of reform, including perfecting hardware facilities, changing function positioning, innovating service models, building a collaborative platform, and building a dynamic mechanism [23]. Each stage of reform has promoted the provision of valuable contracted services by family doctors. This study examined the factors that affected family doctors' motivations to provide contracted services and particularly to act as gatekeepers.

The study found that, to a large extent, family doctors' understanding (cognition) of their own work determined their behavioral orientation and service effectiveness. As the understanding of their work has improved, family doctors have been able to attract patients to sign and renew contracts through comprehensive measures such as medical insurance payments and the provision of quality contracted services. In addition to quality referral resources, family doctors also realize the importance of building partnerships through enhanced communication. The focus of family doctor contracted services has shifted from quantity to service quality and satisfaction [24, 25]. At the same time, family doctors' sense of identification with their profession has improved, especially for professional status, work value, and sense of achievement. The community health service and family doctor system would therefore be expected to be more positive and optimistic. Residents' identity and satisfaction with family doctors have a significant correlation with family doctors' own professional identity and behavior changes [26, 27]. Family doctors have a clear understanding of how to improve residents' perceptions of doctors, by improving their capabilities, service levels, and communication to build partnerships [28, 29].

Increasing the working income of family doctors would help to enhance their job stability and improve the performance of contracted services. It may therefore be necessary to intervene via external factors to improve the motivation of family doctors and therefore increase the provision of contracted services. The market plays a decisive role in resource allocation, and a new service model has been built around contracted services with family doctors as the core, with autonomy over service provision, platform resources, team management, assessment allocation in general service teams, improved job appeal, and promotion prospects [30, 31]. It may also be helpful to establish a performance evaluation and allocation mechanism for medical insurance to pay service fees per capita for contracts, thereby, encouraging and guiding effective contracting [32, 33]. This would form a competition mechanism between different teams providing contracted services. Indicators such as the rate of on-site visits, appointment fulfillment rates, and referral rates could be used in assessments to strengthen the exchange of rights and responsibilities in the contract.

It is also particularly important to broaden the sources of contracted service fees in practice. At present, there are three sources of contracted service fees: medical insurance funds, basic public health service funds, and the contracted residents' payments. Some recommended methods have been used to broaden financing channels for contracted service fees; for instance, medical insurance funds have been directly paid to the contracted team for the contracted service fee, which was not included in the contracted service package fee and did not contribute to the total performance salary of the medical institution. Likewise, the package payment method of medical insurance involves setting the medical insurance payment limit in the personalized contract service package equivalent to the medical insurance funds corresponding to the basic medical care in the personalized contract service package, according to the actual number of contracted people in a certain period by month or quarter. Moreover, the fee standard for contracted service fees of family doctors should be calculated reasonably according to the actual situation of the region. The contracted service fee is a component of the income of family doctors, according to the “Reform of salary system in public hospitals” (allowing medical and health institutions to break through the current wage regulation level and allowing medical service income to deduct costs and withdraw various funds according to regulations), and the requirements for personnel incentives were used for personnel salary distribution. In principle, no less than 70% of the contracted service fee should be paid to the family doctor team, and it should be allocated reasonably according to the results of evaluations such as the number of services, service quality, and residents' satisfaction. The purpose of these methods is to motivate the contracted team.

This study suggests that resolving the policy contradictions associated with the reform of family doctors contracted services is also important. Increased fees for contracted services should break the constraints of the local performance wage ceiling, but the relevant departments must be clear that this has benefits beyond purely financial ones and that it will help to motivate family doctors to provide more valuable services. This study also showed that family doctors who got contracted service fees had higher job recognition and a greater sense of responsibility. The contracted services package should also differentiate between particular patient groups and be dynamically adjusted to enhance the value and attractiveness of family doctor contracted services, while increasing the standardized theoretical and technical training of family doctors and the transfer of specialists. The reform of medical insurance payment methods will establish an incentive mechanism for family doctor contracted services and enable family doctors to increase their income by providing high-quality contracted services. This will be essential to ensuring the sustainable development of the family doctor contracted service system.

The study showed that family doctors were concerned about their physical environment, such as the construction of facilities, as well as their systems and mechanisms. Family doctors' recognition of these operating mechanisms will promote the healthy operation of contracted services, in turn encouraging further reform of the family doctor system. The coordinated support of public hospitals for community contracted services also affects the motivation of family doctors to provide contracted services [34]. To effectively develop the family doctor system, it is necessary to establish a cooperative relationship between different medical institutions, integrate the medical resources of community health service centers and secondary and tertiary hospitals, provide family doctors with additional capacity and technical support, and build a coordinated approach to medical treatment [35]. The signing of contracts to provide family doctor services and the construction of medical consortia are two important starting points for promoting a hierarchical diagnosis and treatment system. These two are in a symbiotic relationship, and we must therefore consider the role of the medical consortium. The establishment of a general practice specialty referral system and enhanced collaboration with community health service institutions should be built into public hospital reform to build a more standardized and efficient community general practice system [36]. In addition, we used structural equation modeling to identify some measures to help improve the technical capabilities of family doctors and open up graded diagnosis and treatment networks, e.g., the fast referral channel platforms to support collaboration with specialists and the training of general practitioners and the information platform to assist in the conduct of remote consultations.

## 5. Conclusions

This study demonstrates that to a large extent, the degree of family doctors' understanding of their work determines their behavioral orientation and service effectiveness. It may be possible to enhance their work stability and improve the performance of contracted services by increasing their income. It is also necessary to intervene in external factors and increase the motivation of family doctors to provide contracted services such as application of artificial intelligence, perfecting the payment method of medical insurance, and improving performance allocation.

## Figures and Tables

**Table 1:** CFA fitting effect of the family doctor measurement model.

Model	CMIN/DF	NFI	CFI	IFI	RMSEA
Reference standard values	2.0–5.0	>0.90	>0.90	>0.90	<0.08
Model 1 (3 factors and 16 indicators)	6.272	0.734	0.763	0.766	0.135
Model 2 (4 factors and 20 indicators)	3.689	0.667	0.725	0.733	0.096
Model R2 (4 factors and 13 indicators)	3.538	0.811	0.854	0.857	0.094

**Table 2:** CFA fitting effect of the dynamic path of the family doctor contracting service model.

Model	*χ* ^2^/d*f*	NFI	IFI	CFI	RMSEA
Reference standard values	2.0–5.0	>0.90	>0.90	>0.90	<0.08
Model 3	3.435	0.685	0.754	0.749	0.092
Model R3	3.837	0.704	0.763	0.758	0.099

**Table 3:** The structural path model' latent variables' standardized effect value.

Independent variables (exogenous/endogenous variables)		Dependent variables (endogenous latent variables)				
		Environmental satisfaction (*η*1)	Income satisfaction (*η*2)	Support satisfaction (*η*3)	Stability (*η*4)	Contracting performance (*η*5)
Cognition (*ξ*_1_)	Direct effect	0.37^*∗*^	0.54^*∗*^	0.25	0.40^*∗*^	0.11
	Indirect effect	**—**	**—**	**—**	0.16	0.26
	Total effect	0.37^*∗*^	0.54^*∗*^	0.25	0.56^*∗*^	0.37
Environmental satisfaction (*η*1)	Direct effect	**—**	**—**	**—**	0.12	**—**
	Indirect effect	**—**	**—**	**—**	**—**	**—**
	Total effect	**—**	**—**	**—**	0.12	**—**
Income satisfaction (*η*2)	Direct effect	**—**	**—**	**—**	0.22^*∗*^	**—**
	Indirect effect	**—**	**—**	**—**	**—**	**—**
	Total effect	**—**	**—**	**—**	0.22^*∗*^	**—**
Stability (*η*4)	Direct effect	**—**	**—**	**—**	**—**	0.51^*∗*^
	Indirect effect	**—**	**—**	**—**	**—**	**—**
	Total effect	**—**	**—**	**—**	**—**	0.51^*∗*^
SMC (*R*^2^)		0.140	0.290	0.062	0.369	0.343

^*∗*^ *P* < 0.05.

## Data Availability

The data used to support this study are available from the corresponding authors upon request.

## Abbreviations

SEM: Structural equation modeling
SMC: Squared multiple correlations
CFA: Confirmatory factor analytic.

## Supplementary Materials

Supplementary Materials Questionnaire of family doctor working status in the community health center.
