# Supplementary material for: Structural Equation Modeling Analysis of Factors Influencing Family Doctor Contracted Services Based on Survey Data from Changning District, Shanghai
Source: Evid Based Complement Alternat Med. 2022 Jun 23;2022:2648833. doi: 10.1155/2022/2648833 (PMC9246594; doi:10.1155/2022/2648833)
Supplement: Supplementary Materials — Questionnaire of family doctor working status in the community health center. [file 2648833.f1.doc]

**Questionnaire of family doctor working status in Community Health Centre**

| **Questionnaire Number** | **Community Health Centre** | **Inspector** |
| --- | --- | --- |
|  |  |  |

Dear family doctor friend:

In order to further understand the work situation of the family doctor system reform in our district, we designed this questionnaire. The questionnaire anonymously, our investigation will be strictly confidential. The investigation will take you 10 minutes or so, thank you for your participation.

1. **Basic information**
2. Your gender: ____

A male B. Female

1. Your age: _____years old (born to zero, every birthday long years)
2. Your highest education: ____

A.High school / technical secondary school and below B.Junior college C.Undergraduate D.Master E.Doctor

1. Your title:____

A.No title B.Primary title C.intermediate title D.Deputy senior title E.Senior title

1. Your department:____

A.General consulting department B.Consulting department of traditional Chinese Medicine C.Rehabilitation department D.Preventive health care department E.Medical and other departments

1. You are engaged in clinical diagnosis and treatment work for ____ years, engaged in community health services center for ____ years, engaged in the work of the family doctor for____years.
2. Your employment form:____
3. Official in the series B.Labor dispatch system C.Temporary contract system D.Others____
4. **Working conditions of the family doctor**
5. Do you have a general practitioner certification qualification____

A.Yes B.No

1. Do you pass the standardized training of general practitioners in Shanghai:____ A.Yes B.No
2. What is the main method of signing with the community residents? ____ A. as a unit of the family B. as the unit of the individual C. The proportion of the above methods is the same D. other (please point out)
3. Which is the main type of contracted services?____

A. family doctor + community nurse B. family doctor + preventive health care staff C. family doctor + community volunteer

D. family doctor studio E. family doctor commonwealth F. others (please point out)

12.The number of your team _____________, do you think the equipment is reasonable?

A. Reasonable C. Unreasonable B. Unclear

If this is unreasonable, it is manifested in____: A. Insufficient quantity B. Incomplete structure C. Other (please specify)

1. How long do you work every day?

A. ≤8 hours B. 8-10 hours C. 10-12 hours D. ≥12 hours

1. As a family doctor, how many community residents have you signed up to now?
2. <500 B.500-1000 C.1000-1500 D.1500-2000 E. other (please point out)
3. Are you going well when you sign with community residents?
4. very smooth B. smooth C. general D. less smooth E. not smooth
5. In what way do you usually signning with the community residents?

A. to sign the contract for the management of chronic disease patients in the clinic

B. to provide on-site service contract C. to carry out health education seminars and lectures in the signing D. through the neighborhood residents to sign E. Other

1. Do you usually take the initiative to sign with the residents?
2. most of the residents should put forward the initiative to sign B. general C. most of the patients themselves ask to sign the contract
3. The main measures to attract residents ______

A. Appointment to see the doctor B. offering two or three referral resources C. save costs D. providing health counseling and health education E. Appropriate to relax the restrictions of dispensing F. providing personalized health management G. other (please point out)

19.At present, which is the most important aspect of carrying out the family doctor signing service?

A. to achieve more residents B. to provide more services C. to increase effectively contract rate D. to ensure the quality of service and satisfaction of the contracted residents E. Other

20.What is the most reason of affecting residents' signing?

A. untrust the family doctors’ ability B. worried about the future of medical treatment only in the community C. worried about the referral resources

D. not sure to get those benefits E. Other

21.Do you often take part in the training activities of general practitioners?

A. often(≥1 time per month) B. occasionally(1-2 times per quarter) C. not often(1-2 times half a year) D. never

22.Do you know the specific content of the implementation of the family doctor system?

A. very clear B. clear C. general D. less clear E. not clear

**(3) Cognition of family doctor service**

23.Do you think that your knowledge and ability could meet the needs of the work of the family doctor?

A. Fully capable of B. Basically be able to C. General D. not able to

24.How do you feel as a family doctor?

A. no pressure B. a little pressure C. general D. relatively large pressure E. a lot of pressure

1. Do you have any technical problems in the process of developing basic medical services?
2. often encountered B. occasionally encountered C. basically not meet D. not clear
3. Do you encounter technical problems in process of developing chronic disease management?
4. often encountered B. occasionally encountered C. basically not meet D. not clear
5. What do you think is the most deficient in carrying out the family doctor signing service?

A. health management theory B. clinical experience and skills C. rich specialized medical resources D. ability to communicate with patients E. others (please point out)

1. What is the degree of acceptance and participation of community residents in the family doctor system?
2. very acceptable B. acceptable C. general D. poor E. very poor
3. Compared to the previous, do you think the ability is improved after the family doctor training?
4. significantly improve B. slightly improve C. did not change D. not clear
5. As a family doctor, do you think your ability has been played and displayed?
6. full play B. basic play C. general D. less play E. very difficult to play
7. Compared with the specialist, what are you feeling as a family doctor's professional reputation?
8. higher reputation B. relatively high reputation C. general D. a little low E. very low reputation
9. To 100 points out, what is your self-evaluation as a family doctor?

A. 0-50 points B. 51-60 points C. 61-70 points D. 71-80 points E.81-90 points F.91-100 points

1. To 100 points out, what do you think the family doctor in the work sense of accomplishment and satisfaction is?
2. 0-50 points B. 51-60 points C. 61-70 points D.71-80 points E. 81-90 points F.91-100 points
3. Which option is most likely to give you a sense of accomplishment and satisfaction?

A. high income B. community residents’ and patients' recognition, good reputation C. higher occupational reputation and status D. health improvement E. advanced in deputy, honorary titles and social identity F. other (please specify)

1. As a family doctor, do you think the most important aspect of obtaining the approval of the residents is?

A. qualifications B. age C. title D. service level E. service attitude

F. ability to communicate G. the service community and the level of service objects

1. What is the main reason for the residents not to accept the designated first diagnosis system in the community?
2. worry about misdiagnosis B. worry about delay treatment C. impact of free choice D. others (please point out)
3. Do you think the establishment of the Regional Medical Association is conducive to the implementation of the family doctor system?
4. conducive B. not conducive C. general D. not clear
5. What is the most urgent need in this area to further promote family doctor system?
6. strengthen the policy advocacy to guide the establishment of community health

B. health service centers designated the first diagnosis system

C. the establishment of two-way referral system

D.GP training to improve the service level of the family doctor

E. health information

F. improve the basic drug system

G. the formation of the Medical Association

H. strengthen the performance appraisal, improve the family doctor treatment

I. Other (please specify)

1. What are the most difficulties in the family doctor system? (multiple-choice and sort)

| **Difficulty and worry** | **sort** |
| --- | --- |
| A. residents do not understand the signing service, publicity work is not implemented |  |
| B. residents for family doctors do not trust, do not want to sign |  |
| C. residents of the obligations of the contract has a fear of psychological, unwilling to sign |  |
| D. residents can not be in accordance with the signing of the designated medical and community first diagnosis |  |
| E. its own limited capacity, it is difficult to truly provide residents with valuable services to attract and retain residents signed |  |
| F. Medical Association does not really provide support platform and referral channels |  |
| G. family doctor's allowance or service fee can not be paid in full and timely |  |
| H. relevant supporting measures are not in place |  |

**(4)Job satisfaction**

1. Your average monthly income nearly the three months (after tax, including the performance bonus)?

A. 3000 RMB B. 3000-4999 RMB C. 5000-7999 RMB

D. 8000-9999 RMB E. 10000-15000 RMB F. More than 15000 RMB

1. As a family doctor, what is the additional allowance or service fee account for your income?

A. <10% B. 10-20% C. 20-30% D. 30-40% E. 40-50% F. More than 50%

1. Are you satisfied with the current income level of family doctors?
2. very satisfied B. satisfied C. general D. dissatisfied E. very dissatisfied
3. Do you think the labor remuneration match to the family doctor value?
4. very match B. match C. general D. less match E. not match
5. Are you satisfied with the overall feel benefits provided by the family doctor?
6. very satisfied B. satisfied C. general D. dissatisfied E. very dissatisfied
7. Compared with the doctor, do you think the family doctor's income and pay is fair?

A. very fair B. fair C. less fair D. not fair

1. Do you feel the enthusiasm of the units of quantity and quality of the performance evaluation can mobilize the family doctor?
2. very obvious B. obvious C. general D. less obvious E. not obvious
3. Are you satisfied with the two or three level hospital doctors and you to cooperate to support the diagnosis and treatment services?
4. very satisfied B. satisfied C. general D. dissatisfied E. very dissatisfied
5. Is your community (neighborhood) support satisfied with your cooperation?
6. very satisfied B. satisfied C. general D. dissatisfied E. very dissatisfied
7. Are you satisfied with the support of your team members?
8. very satisfied B. satisfied C. general D. dissatisfied E. very dissatisfied
9. The following project are various environmental factors, please answer to each item.

| Number | Project | Very satisfied | Satisfied | Commonly | Dissatisfied | Very dissatisfied |
| --- | --- | --- | --- | --- | --- | --- |
| A. | Your office conditions and space |  |  |  |  |  |
| B. | The organization and equipment conditions |  |  |  |  |  |
| C. | Unit of medical support system |  |  |  |  |  |
| D. | Administrative support system |  |  |  |  |  |
| E. | The organization and management |  |  |  |  |  |
| F. | With the people and the promotion system |  |  |  |  |  |
| G. | The central leadership quality and management ability |  |  |  |  |  |
| H. | Interpersonal relationships of unit colleagues |  |  |  |  |  |
| I. | Family doctor information means |  |  |  |  |  |

Thank you again for your great cooperation!!!
